# Spontaneous Remission of Isaacs’ Syndrome

**DOI:** 10.4274/balkanmedj.galenos.2019.2019.2.53

**Published:** 2019-07-11

**Authors:** Sadettin Uslu, Tuba Yüce İnel, Ali Karakaş, Fatoş Önen

**Affiliations:** 1 Department of Rheumatology, Internal Medicine, Dokuz Eylül University School of Medicine, İzmir, Turkey

An 18-year-old man presented with generalized body ache, muscle cramps, weight loss, and painful muscle twitching in his lower extremities. His symptoms aggravated with physical exertion. There was an increased sweating on his limbs and trunk. His medical history was notable for lichen planus. He was a nonsmoker and did not use illicit drugs. Family history was insignificant. On examination, fasciculations on his lower extremities were observed (Video 1).

Consciousness, mental state, tone, power, reflexes, senses, cerebellar signs, and cranial nerves on examination were normal. Laboratory investigations of the patient revealed elevated muscle enzymes: creatine phosphokinase as 922 U/L (0-171 U/L), aspartate aminotransferase as 85 U/L (0-50 U/L), and alanine aminotransferase as 157 U/L (0-50 U/L). Serum electrolytes, thyroid hormone, calcium, phosphate, and vitamin D levels were normal. The expression of anti-nuclear, antineutrophil cytoplasmic, and extractable nuclear antibodies was negative. Magnetic resonance imaging showed no evidence of myositis, and the cranial magnetic resonance imaging was normal. A written informed consent was obtained from the patient.

Nerve conduction studies revealed normal electrophysiological findings. However, a decremental response to repetitive nerve stimulation was detected. Needle electromyography examination revealed spontaneous motor unit potential transitions and fasciculations in the gastrocnemius muscle (Table 1). The concentration of serum anti-voltage-gated potassium channel antibodies was 144 pmol/L (n<85 pmol/L). Computerized tomography scan of the chest and abdomen did not show any evidence of malignancy. Based on the clinical and electrophysiological findings and an antibody positivity, a diagnosis of Isaacs’ syndrome was made. While further investigations were in progress, the patient recovered spontaneously without treatment.

Isaacs’ syndrome is a rare neuromuscular disorder, characterized by hyperexcitability of the peripheral nerves and continuous activation of muscle fibers, due to auto-antibodies directed against the voltage-gated potassium channels (1). In case of prolonged painful muscle cramps or myokymia, excessive sweating, pseudohypertrophy on examination, and findings of increased muscle enzymes; a diagnosis of Isaacs’ syndrome should be considered. Patients with Isaacs’ syndrome usually require symptomatic treatment with anticonvulsants such as carbamazepine and phenytoin. Moreover, in refractory cases, immunosuppressive therapy including corticosteroids, with or without azathioprine, could be considered. Furthermore, some patients may benefit from plasma exchange or intravenous immunoglobulin therapy (2). Although spontaneous remission is rare, it could be observed in some patients in the course of the disease (3).

**Video 1.** The video shows the fasciculations of lower extremity.

**(10.4274/balkanmedj.galenos.2019.2019.2.53.video.1)**

## Figures and Tables

**Table 1:**
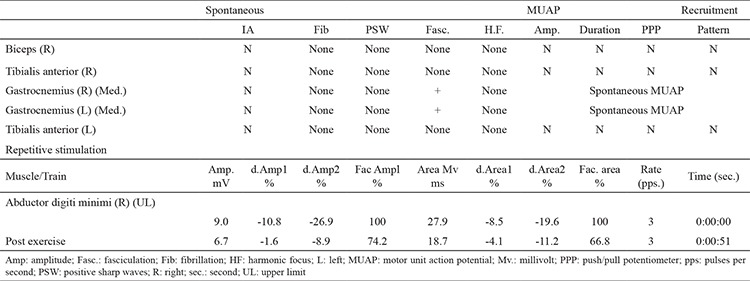
Electromyography results
